# Novel Configurations of Ultrahigh Frequency (≤600 MHz) Analog Frontend for High Resolution Ultrasound Measurement

**DOI:** 10.3390/s18082598

**Published:** 2018-08-08

**Authors:** Min Gon Kim, Jinhyoung Park, Qifa Zhou, Koping Kirk Shung

**Affiliations:** 1 Department of Biomedical Engineering, University of Southern California, Los Angeles, CA 90089, USA; mingonki@usc.edu (M.G.K.); qifazhou@usc.edu (Q.Z.); kkshung@usc.edu (K.K.S.); 2 Department of Biomedical Engineering, Sungkyunkwan University, Suwon, Gyeonggi-do 16419, Korea

**Keywords:** ultrahigh frequency ultrasound, Golay coded excitation, high resolution imaging

## Abstract

In this article, an approach to designing and developing an ultrahigh frequency (≤600 MHz) ultrasound analog frontend with Golay coded excitation sequence for high resolution imaging applications is presented. For the purpose of visualizing specific structures or measuring functional responses of micron-sized biological samples, a higher frequency ultrasound is needed to obtain a decent spatial resolution while it lowers the signal-to-noise ratio, the difference in decibels between the signal level and the background noise level, due to the higher attenuation coefficient. In order to enhance the signal-to-noise ratio, conventional approach was to increase the transmit voltage level. However, it may cause damaging the extremely thin piezoelectric material in the ultrahigh frequency range. In this paper, we present a novel design of ultrahigh frequency (≤600 MHz) frontend system capable of performing pseudo Golay coded excitation by configuring four independently operating pulse generators in parallel and the consecutive delayed transmission from each channel. Compared with the conventional monocycle pulse approach, the signal-to-noise ratio of the proposed approach was improved by 7–9 dB without compromising the spatial resolution. The measured axial and lateral resolutions of wire targets were 16.4 µm and 10.6 µm by using 156 MHz 4 bit pseudo Golay coded excitation, respectively and 4.5 µm and 7.7 µm by using 312 MHz 4 bit pseudo Golay coded excitation, respectively.

## 1. Introduction

There have been several studies on accurately visualizing living tissue with minimal effects on normal physiology and structures with ultrasound, inaudible sound having the frequency range over 20 kHz and transmitted or detected by transducers employing piezoelectric materials [1,2,3,4]. By increasing the operational frequency of ultrasound technologies, the improved image resolution enables the exploration for the precisely quantitative indicator of arteriosclerosis using intravascular ultrasound (IVUS) imaging, highly detailed images of outer layers of skin, anterior segment of the eye, and measurement of the mechanical properties of small objects [5,6,7,8,9]. For the cellular applications, a working frequency range at least over 100 MHz is required to achieve the adequate acoustic beam size similar to the typical cell size [10,11,12]. However, as the higher frequency ultrasound exhibits greater attenuation than the lower frequency ultrasound, the increased signal loss was observed and thus the signal-to-noise ratio (SNR), the ratio of signal power to the noise power, was inherently sacrificed. For these reasons, commercially available ultrasound imaging systems in the very high frequency (VHF) range between 30 MHz and 300 MHz and ultrahigh frequency (UHF) range from 300 MHz to 3 GHz have been elevating the transmit voltage level for enhancing the SNR. However, this may cause damages on the extremely thin piezoelectric layer within the VHF and UHF transducers [13]. In order to simultaneously satisfy two respects including adequate improving SNR and protecting such thin piezoelectric transducers, it is necessary to take steps to use coded excitation approach.

Coded excitation techniques have been widely used to achieve higher SNR without degradation of axial resolution compared to conventional pulse generation methods [14,15,16]. In previous studies, O’Donnell reported 15 dB SNR improvements in medical ultrasound frequency range and Hu demonstrated 14 dB SNR improvements in the high frequency range [17,18]. Thus, the coded excitation technique is well-known for enhancing the SNR in such frequency ranges; however, the device for VHF coded excitation would only be available in combination with bench top devices which delivers transmit voltage much less efficiently and does not effectively block external noise source with several cable connections, which may lead to poor SNR. Moreover, UHF analog frontend system with coded exaction is even not currently available in the market.

In order to address this problem, we present a prototype of 4 channel UHF ultrasound analog frontend module capable of performing coded excitation imaging for the enhancement of the SNR without elevating the transmit voltage level. We initially developed a single channel frontend module consisting of a frequency tuneable pulse generator and an analog receiver which works over VHF and UHF from 100 MHz to 600 MHz. Then, we configured four single channel modules in parallel with each other in order to generate two types of pseudo Golay coded excitations to improve SNR for the measurement. Since the typical UHF mono cycle pulse transmitter is unable to transmit inversed waveforms required for generating the conventional Golay code, pseudo Golay code which employs 0 V for the bipolar pulse generator and 180-degree phase shifted pulse for the unipolar pulse generator instead of using the inversed waveform is proposed, and the echo signal is compressed with a mismatched filter. The performance of the developed system was verified by using two different ultrasonic transducers having different working frequency ranges at VHF and UHF for imaging wire targets in terms of SNR and the spatial resolution.

## 2. Materials and Methods

### 2.1. Design and Development of the Monocycle Pulse Generator

**Figure 1:**
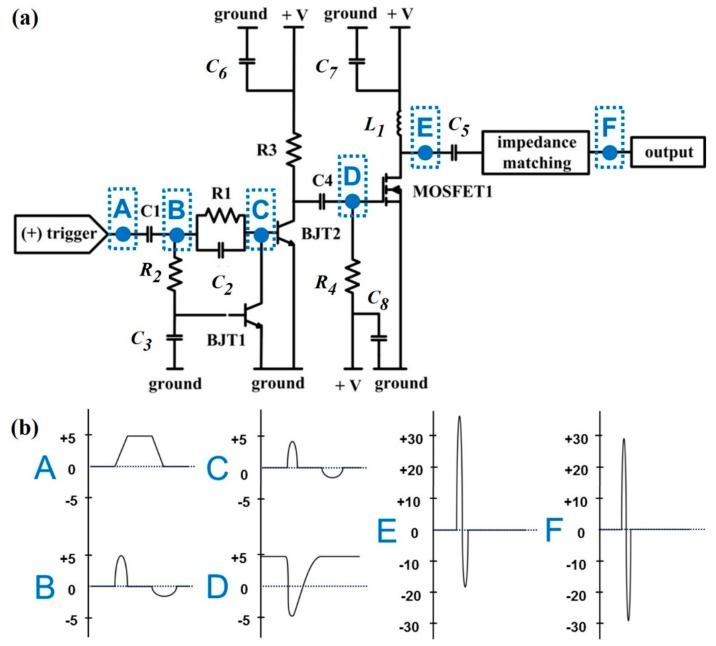
Proposed pulse generator: (**a**) Schematics of pulse generator; (**b**) output wave forms at the blue dotted position from A to F. The unit of the *y*-axis is Volts.

The monocycle pulse generator consisted of a pulse forming network [19] and an amplifier utilizing radio frequency laterally diffused metal oxide semiconductor (RF LDMOS) technology. Figure 1a shows the schematics and Figure 1b depicts the output signal of each stage from A to F. The pulse forming network included a timing circuit (*R*_2_, *C*_3_), a speed-up capacitor (*C*_2_), and switching bipolar transistor (BJT1). When a single 5 V rectangular unipolar pulse trigger signal was received at the input port, the voltage at the base of BJT2 (NE46100, California Eastern Laboratories, Santa Clara, CA, USA) reached the threshold level for being turned on. To sharpen the edges of the pulse generated, a speed up capacitor *C*_2_ was located before the base of BJT2. Most transistors had a delay time in turning on and storage time in shutting off. These two parameters could be adjusted to minimize the transitional time of BJT2 and eventually the pulse width. The storage time depended on the amount of current injected into the base of BJT2 and the additional current to maintain the saturated condition. The delay time could be shortened by draining the base current quickly with a strong reverse bias. The network of *R*_1_ and *C*_2_ accomplished that purpose. During the voltage rise, *R*_1_ was shorted by *C*_2_, minimizing the time to reach 5 V by injecting the current into the base of BJT2 and reducing the supply current for holding the voltage. *C*_2_ was then shorted out during the falling edge of the input signal. The values of *R*_1_ and *C*_2_ were set to 100 Ω and 47 pF by assuming the rise time needed to be at least two times faster than the targeted output frequency of 100 MHz. Meanwhile, when the trigger pulse was applied to the input A (Figure 1b), the capacitor in the timing network (*C*_3_) started to be charged. Once the voltage on *C*_3_ approached the threshold voltage for turning on BJT1 (NE46100, California Eastern Laboratories, Santa Clara, CA, USA), BJT1 drained the current from point C (Figure 1b) to the ground, and BJT2 was shut off. Therefore, the combination of the timing circuit and speed-up capacitor allowed for the generation of a short pulse out of the low frequency trigger signal. The on time of BJT1 was dependent on the RC constant shown in the equation below and could be controlled by changing the resistance value of *R*_2_ [20].

(1) $$U_{C3}=U_{B}\cdot (1-e^{-\frac{time}{R_{2}\cdot C_{3}}})$$

where *U*_C3_ is the base voltage, *U*_B_ is the voltage at point B (Figure 1b) and *time* is the on time of BJT1. The values of *R*_2_ and *C*_3_ for generating the output pulse over 100 MHz were 500 Ω and 4 pF.

The following stage of the proposed pulse generator was an amplifier utilizing RF LDMOS that has a narrow channel length and a heavily doped n-type region for obtaining high output current and minimal on-resistance [21]. The output of the pulse forming network at point D (Figure 1b) was a pulse having a negative voltage peak which turned on MOSFET1 (BLF571, NXP Semiconductors, Eindhoven, The Netherlands). The output frequency of the common source power amplifier was tuned by setting L_1_ and C_5_ to 27 nH and 47 pF for the 100 MHz pulse output, 27 nH and 10 pF for the 200 MHz pulse output, 25 nH and 10 pF for the 300 MHz pulse output, and 3 nH and 9 pF for the 500 MHz pulse output, respectively. The output impedance of the amplifier was matched with 50 Ω by placing a 50 Ω resistor in parallel. 

### 2.2. Design and Development of 4-Bit Pseudo Golay Coded Excitation Generator

**Figure 2:**
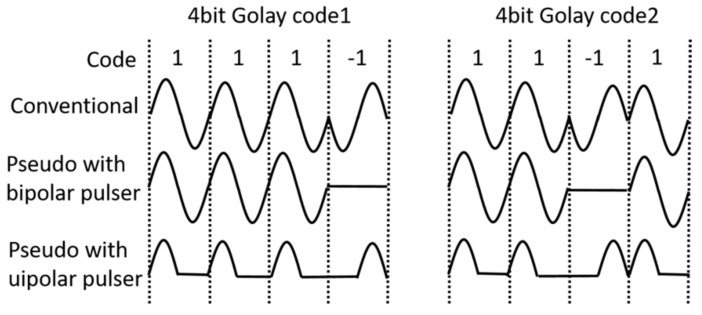
Comparison of waveforms of the conventional, pseudo Golay code with bipolar and unipolar pulses.

The proposed phase-modulated waveform generator comprised four identical ultrahigh frequency monocycle pulse generators aligned in parallel with each other. The trigger inputs for each pulse generators were applied independently for controlling the pulse generators separately. The outputs from those generators were merged into the same output load. Multiple types of waveforms, e.g., mono cycle, dual cycle, quad cycle, phase-modulated burst, could be generated by controlling the triggering time for each pulse generator. For example, mono cycled pulse could be generated by triggering the pulse generators in a same time, and the four cycled burst can be made by triggering one pulse generators after another one by setting the delay time of the single cycle burst. Dual cycle burst can be generated by triggering two pulse generators together after another two. In implementing the conventional phase-modulated signals with the binary code composed of ‘1’ and ‘−1’, e.g., Golay coded excitation, a single cycled burst or two represents ‘1’, and its inverted form does ‘−1’. Since the developed pulse generator could not invert the waveform, pseudo Golay code defines ‘−1’ with zero voltage for bipolar pulse generator mode or with half-cycle-shifted unipolar burst for the unipolar pulse generator mode shown in the Figure 2.

**Figure 3:**
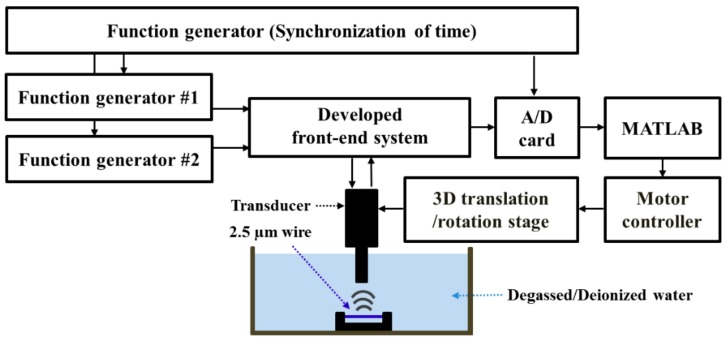
High frequency mechanical ultrasound system setup.

**Figure 4:**
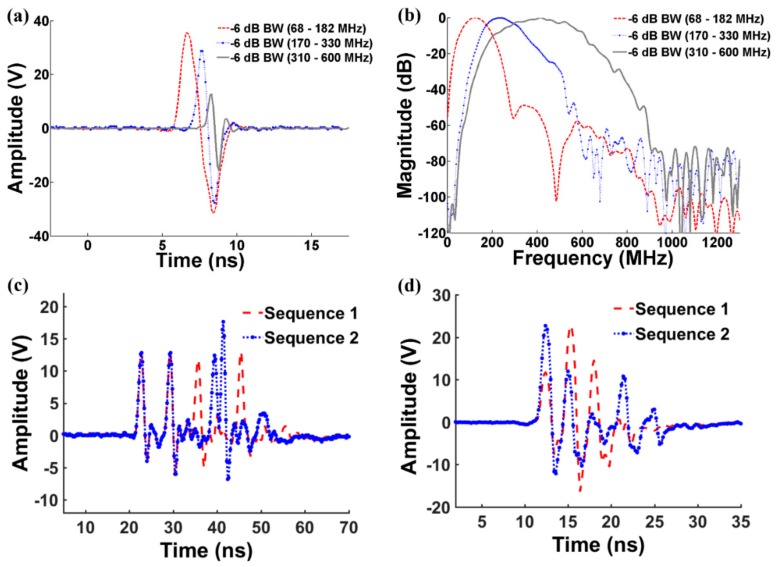
Measured output pulses from the proposed transmitter: (**a**) Output pulses in the time domain; (**b**) output pulses in the frequency domain; (**c**) four-bit 156 MHz unipolar pseudo Golay codes from the combined pulse generators; and (**d**) four-bit 312 MHz bipolar pseudo Golay codes from the combined pulse generators.

In the experimental setup, the output signals generated by two channel function generators #1 and #2 in Figure 3 utilized for trigger pulses were synchronized by function generator #3. Then, we controlled time delay (6.4 ns for 156 MHz and 3.2 ns for 312 MHz) by using the function generators #1 and #2. For the generation of 4 bit 156 MHz unipolar Golay code (Figure 4c), sequence 1 with the dotted line and sequence 2 with the solid line were generated, and the waveforms are comparable with the ones shown in Figure 2. For the generation of 4 bit 312 MHz bipolar Golay code (Figure 4d), sequence 1 and sequence 2 were generated. For the sequence 1, the first, the second, the third, and the fourth pulses were generated at 11.2 ns, 14.4 ns, 17.6 ns, and 20.8 ns. The echo signals of the pseudo Golay coded transmit are compressed with the mismatched filter which employs the conventional Golay waveforms shown in Figure 2.

### 2.3. Experimental Setup for Performance Evaluation

Two single element lithium niobate (LiNbO_3_) ultrasonic transducers were custom-made [22,23] and used for the performance evaluation. The center frequency, −6 dB bandwidth, and f-number of the VHF transducer were 116 MHz, 28%, and 0.7, respectively, and those values for UHF transducer were 319 MHz, 34%, and 1.3, respectively.

The designed pulse generator performing 4-bit pseudo Golay code was integrated into an ultrasound biomicroscope (UBM) which mechanically translated an ultrasonic transducer over imaging targets as shown in Figure 3. While the transducer was moving with a step distance of 1 µm over 2.5 µm tungsten wires, trigger pulses from the function generators #1 and #2 (AFG 3252, Tektronix, Beaverton, OR, USA) for the input to the monocycle pulse generator with the pre-defined delay time were transmitted in a 20 kHz pulse repetition frequency (PRF) generated by function generator #3 (33250A, Agilent, Santa Clara, CA, USA). The trigger pulses delivered transmit waveforms with transmitter voltage of 20 V_pp_~30 V_pp_ to unipolar 156 MHz and bipolar 312 MHz ultrasound transducers. The received echo signal is amplified using a custom-built receiver amplifier with an amplification gain of 19.5 dB and a noise figure of 1.6 dB measured by a spectrum analyzer (E4402B, Agilent technologies, Santa Clara, CA, USA) and digitized in a 1 GHz sampling rate with a commercial digitizer (RazorMax 16, GaGe, Lockport, IL, USA). To compare the performance evaluation, a commercial pulser/receiver (Panametrics 5900, Panametrics, Inc., Waltham, MA, USA) was used to excite the transducers with the electrical pulse having the energy level of 1 µJ (~100 V_pp_) and the received echo signal was amplified by 20 dB. The analog high pass filter of 1 MHz and low pass filter of 200 MHz were set in the pulser/receiver. The comparison of the performance between the proposed pulser/receiver and the commercial pulser/receiver was summarized in Table 1. The digitized signal was accumulated in the memory of a computer and processed with a custom-built Matlab Program. For the 156 MHz imaging frequency, the performance evaluation was conducted by three types of transmit waveforms including impulses from the commercial pulser/receiver, unipolar single pulses and 4-bit pseudo Golay coded excitation from the implemented pulser/receiver. Also, for the 312 MHz imaging frequency, performance testing was evaluated with two types of transmit waveforms such as a bipolar single pulse from the implemented pulser/receiver and 4 bit pseudo unipolar Golay coded excitations.

## 3. Results

### 3.1. Performance Measurements of the Developed Pulse Generator

Figure 4 demonstrates the measured output pulses from the implemented pulse generator. Figure 4a shows the output pulse waveforms and Figure 4b shows the frequency responses for three different frequency ranges. The center frequencies of the provided signals are 125 MHz, 250 MHz, and 455 MHz and the -6 dB bandwidths are 68–182 MHz, 170–330 MHz, and 310–600 MHz with peak-to-peak voltage levels of 70 V_pp_, 60 V_pp_, and 30 V_pp_, respectively. It was found that the pulse repetition frequency (PRF) can be increased up to 1 MHz with a 100 MHz single cycle of 70 V_pp_ which corresponds to 6 W output power. Figure 4c,d demonstrate 4 bit 156 MHz unipolar Golay codes from the combined pulse generators and 4 bit 312 MHz bipolar Golay codes from the combined pulse generators, respectively. The phase modulation was well identified in the figures. Also, three consecutive cycled sinusoidal burst which could be used for color or Doppler imaging mode (Figure 4d).

### 3.2. Performance Evaluation of 4 Bit Golay Code in Wire Target Study

**Figure 5:**
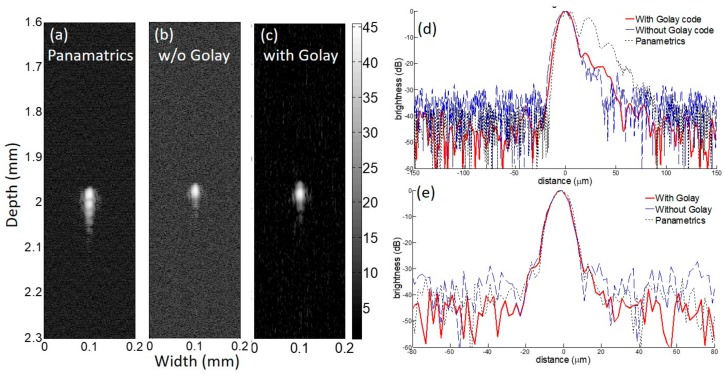
Wire target study results at 156 MHz imaging frequency: B-mode images are acquired with (**a**) Panamatrics pulser/receiver; (**b**) proposed analogue frontend without coded excitation; and (**c**) with coded excitation. The brightness profiles in dB scale represents (**d**) axial profile and (**e**) lateral profile. Note that the red solid, blue dashed, and black dotted lines represent the profiles with Golay, without Golay, and Panametrics pulser/receiver, respectively. Note that the scale of the gray bar is decibel (dB).

**Figure 6:**
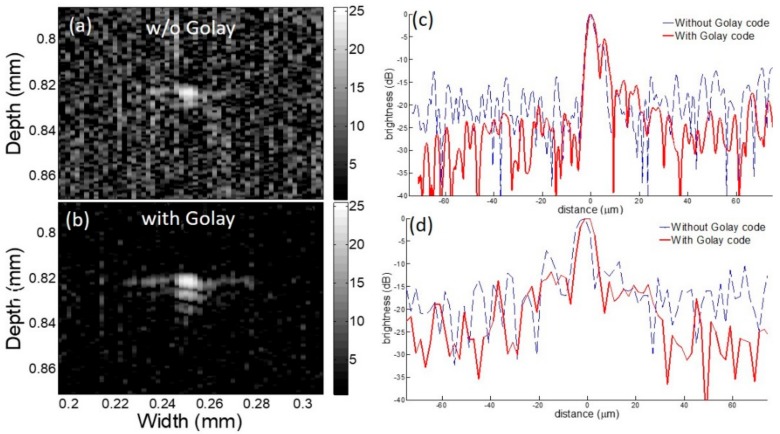
Wire target study results with 312 MHz imaging frequency: B-mode images are acquired with the proposed analogue frontend (**a**) without coded excitation and (**b**) with coded excitation. The brightness profiles in the dB scale represent (**c**) axial profile and (**d**) lateral profile. Note that the red solid and blue dashed lines represent the profiles with Golay and without Golay, respectively. Note that the scale of the gray bar is decibel (dB).

Figure 5 and Figure 6 demonstrates the wire target study results acquired by 156 MHz and 312 MHz imaging frequencies, respectively. In Figure 5, the results from three types of transmit waveforms; impulse from the commercial Panametrics pulser/receiver, unipolar single pulse from the implemented pulser/receiver and 4 bit pseudo Golay coded excitation are compared. The −6 dB axial resolution values for the impulse, unipolar, and Golay code are 18.1 µm, 17.2 µm, and 16.5 µm, respectively and the lateral resolution values are 11.5 µm, 9.9 µm, and 10.0 µm, respectively. The SNR for the impulse, unipolar, and Golay code are 46 dB, 40 dB, and 47 dB, respectively.

In Figure 6, the results from two types of transmit waveforms; a bipolar single pulse from the implemented pulser/receiver and 4-bit pseudo unipolar Golay coded excitation are compared. The −6 dB axial resolution values for the bipolar and the Golay transmissions are 4.4 µm and 4.9 µm, respectively and the lateral resolution values are 7.6 µm and 7.2 µm, respectively. The SNR for the single cycle and Golay transmission are 20 dB and 29 dB, respectively. 

## 4. Discussions

In the previous study, VHF/UHF ultrasound imaging experienced difficulty in getting a decent SNR, since the transmitter in the analogue frontend mostly employed benchtop mono cycle pulse generators or a combination of commercial function generators and an RF power amplifier [19]. Here, the implemented frontend could perform multiple cycled bursts that were essential for those imaging modes and could also generate Golay coded excitation for enhancing SNR by more than 7 dB without elevating the transmit voltage level, as demonstrated in Figure 5 and Figure 6. The improved SNR with high resolution of a few micrometers was accomplished by the integrated pulser and the receiver in a board capable of performing coded excitation. To the best of our knowledge, this is the first work that shows 4 channel frequency tunable pulse generators for coded excitation and a custom-built analog receiver on a single board which works over VHF and UHF from 100 MHz to 600 MHz.

In the UHF range, the maximum output voltage level from each channel is around 20 V_pp_ less than the level of 1 μJ (~100 V_pp_) from the commercial impulse transmitter. The lower transmit voltage will cause the decreased SNR caused by the lower transmit voltage level compared with the commercial product was overcome by employing UHF coded excitation resulting in even 1 dB improvement in SNR shown in the wire target study. Although the lower voltage level will be helpful in protecting UHF ultrasound transducers, the elevation of the higher transmit voltage level may be required to scan biological samples. As a future work, the increase in the number of channels may be the one option to enhance SNR by configuring the extra channels for increasing the transmit voltage level with the use of multiple channels for forming each sinusoidal cycle in the Golay code or elongating the length of the Golay code. The use of coded excitation with the proposing frontend configuration will allow high definition imaging without compromising SNR and the safe operations of VHF or UHF ultrasound transducers.

By using the proposed approach, the improved SNR is achieved without a loss in spatial resolution. The echo signal from the pseudo Golay transmission seems to be well compressed by the use of a mismatched filter since the measured spatial resolution with the Golay code is quite similar to that with the mono cycled bursts. Thus, we believe that this experimental result is a prominent stepping stone for high definition imaging with the resolution of a few microns on 2D or 3D brightness mode. Also, the device is applicable to the functional mode such as color, Doppler mode without the aid of noisier benchtop pulse generators due to its capability of generating multi-cycled sinusoidal bursts. Another remarkable characteristic of the system is the pulsing of waveforms at high PRF up to 1 MHz without degrading the output voltage level, which can be used for monitoring the very fast translation of living tissue/cells in response to the acoustic radiation force, which cannot be achieved by lower PRF [9,12].

## 5. Conclusions

The design of UHF ultrasound analog frontend system with pseudo Golay coded excitation sequence for high resolution imaging applications was presented in this paper. Our approach combines multiple sets of UHF pulse generators and the application of the time delay to each transmission enabled the arbitrary phase-modulated signals. The SNR of the proposed approach was improved by 7–9 dB with 4-bit pseudo Golay code compared to the conventional monocycle pulse method. With the proposed approach, the enhancement of SNR is achieved by using mismatched filters, without a loss in spatial resolution. These experimental results have shown that the developed UHF ultrasound analog frontend system can be used for the visualization of micron-scale biological sample structures with high resolution without damaging the ultrasonic transducer. 

## Figures and Tables

**Table 1:** Performance comparison between the proposed pulser/receiver and the commercial pulser/receiver.

	Proposed Pulser/Receiver	Commercial Pulser/Receiver (Panametrics 5900PR)
Transmitter voltage	20 V_pp_~70 V_pp_	1 µJ (~100 V_pp_)
Number of transmitter channel	1~4	1
Implementation of coded excitation	O	X
Maximum bandwidth	600 MHz	200 MHz
Maximum pulse repetition frequency (PRF)	1 MHz	20 kHz
Maximum receiver gain (Receiver gain used for experiments)	60 dB (19.5 dB)	54 dB (20 dB)

## References

[1] Liang H.D., Blomley M.J. (2003). The role of ultrasound in molecular imaging. Br. J. Radiol..

[2] Shung K.K. (2009). High frequency ultrasonic imaging. J. Med. Ultrasound.

[3] Lockwood G.R., Turnbull D.H., Christopher D.A., Foster F.S. (1996). Beyond 30 MHz: Applications of high frequency ultrasonic imaging. IEEE Eng. Med. Biol. Mag..

[4] Foster F.S., Pavlin C.J., Harasiewicz K.A., Christopher D.A., Turnbull D.H. (2000). Advances in ultrasound biomicroscopy. Ultrasound Med. Biol..

[5] Lee J., Moon J.Y., Chang J.H. (2018). A 35 MHz/105 MHz dual-element focused transducer for intravascular ultrasound tissue imagingu the third harmonic. Sensors.

[6] Yoon S., Kim M.G., Williams J.A., Yoon C., Kang B.J., Cabrera-Munoz N., Shung K.K., Kim H.H. (2015). Dual element needle transducer for intravascular ultrasound imaging. J. Med. Imaging.

[7] Knspik D.A., Starkoski B., Pavlin C.J., Foster F.S. (2000). A 100–200 MHz ultrasound biomicroscope. IEEE Trans. Ultrason. Ferroelectr. Freq. Control.

[8] Silverman R.H., Lizzi F.L., Ursea B.G., Cozzarelli L., Ketterling J.A., Deng C.X., Folberg R., Colemen D.J. (2001). Safety levels for exposure of cornea and lens to very high-frequency ultrasound. J. Ultrasound Med..

[9] Park J., Lee J., Lau S.T., Lee C., Huang Y., Lien C.-L., Shung K.K. (2012). Acoustic Radiation Force Impulse (ARFI) Imaging of Zebrafish Embryo by High-Frequency Coded Excitation Sequence. Ann. Biomed. Eng..

[10] Hwang J.Y., Lee C., Lam K.H., Kim H.H., Lee J., Shung K.K. (2014). Cell membrane deformation induced by a fibronectin-coated polystyrene microbead in a 200-MHz acoustic trap. IEEE Trans. Ultrason. Ferroelectr. Freq. Control.

[11] Yoon S., Kim M.G., Chiu C.T., Hwang J.Y., Kim H.H., Wang Y., Shung K.K. (2016). Direct and sustained intracellular delivery of exogenous molecules using acoustic-transfection with high frequency ultrasound. Sci. Rep..

[12] Kim M.G., Park J., Lim H.G., Yoon S., Lee C., Chang J.H., Shung K.K. (2017). Label-free analysis of the characteristics of a single cell trapped by acoustic tweezers. Sci. Rep..

[13] Kim M.G., Yoon S., Kim H.H., Shung K.K. (2016). Impedance matching network for high frequency ultrasonic transducer for cellular applications. Ultrasonics.

[14] Misaridis T.X., Pedersen M.H., Jensen J.A. Clinical use and evaluation of coded excitation in B-mode images. Proceedings of the 2000 IEEE International Ultrasonics Symposium.

[15] Chiao R.Y., Hao X. (2005). Coded excitation for diagnostic ultrasound: A system developer’s perspective. IEEE Trans. Ultrason. Ferroelectr. Freq. Control.

[16] Oelze M.L. (2007). Bandwidth and resolution enhancement through pulse compression. IEEE Trans. Ultrason. Ferroelectr. Freq. Control.

[17] O’Donnell M. (1992). Coded excitation system for improving the penetration of real-time phased-array imaging systems. IEEE Trans. Ultrason. Ferroelectr. Freq. Control.

[18] Hu C.H., Liu R., Zhou Q., Yen J., Shung K.K. (2006). Coded excitation using biphase-coded pulse with mismatched filters for high frequency ultrasound imaging. Ultrasonics.

[19] Kim M.G., Choi H., Kim H.H., Shung K.K. Bipolar pulse generator for very high frequency (>100 MHz) ultrasound applications. Proceedings of the 2013 IEEE International Ultrasonics Symposium.

[20] Oldham K.B. (2004). The RC time “constant” at a disk electrode. Electrochem. Commun..

[21] Sung K., Won T. (2011). Design of a 100 V high-side n-channel LDMOS transistor for breakdown voltage Enhancement. J. Korean Phys. Soc..

[22] Cannata J.M., Ritter T.A., Chen W.H., Silverman R.H., Shung K.K. (2003). Design of efficient, broadband single-element (20–80 MHz) ultrasonic transducers for medical imaging applications. IEEE Trans. Ultrason. Ferroelectr. Freq. Control.

[23] Lam K.H., Ji H.F., Zheng F., Ren W., Zhou Q., Shung K.K. (2013). Development of lead-free single-element ultrahigh frequency (170–320 MHz) ultrasonic transducers. Ultrasonics.

